# Fabrication and Performance of MEMS-Based Pressure Sensor Packages Using Patterned Ultra-Thick Photoresists

**DOI:** 10.3390/s90806200

**Published:** 2009-08-05

**Authors:** Lung-Tai Chen, Jin-Sheng Chang, Chung-Yi Hsu, Wood-Hi Cheng

**Affiliations:** 1 Micro-System Technology Center, Industrial Technology Research Institute. 709 Tainan, Taiwan; E-Mails: rexchen@itri.org.tw (L.T.C.); jimmychang@itri.org.tw (J.S.C.); enoshhsu@itri.org.tw (C.Y.H.); 2 Institute of Electro-Optical Engineering, National Sun Yat-Sen University, 804 Kaohsiung, Taiwan

**Keywords:** pressure sensor, photoresist, packaging

## Abstract

A novel plastic packaging of a piezoresistive pressure sensor using a patterned ultra-thick photoresist is experimentally and theoretically investigated. Two pressure sensor packages of the sacrifice-replacement and dam-ring type were used in this study. The characteristics of the packaged pressure sensors were investigated by using a finite-element (FE) model and experimental measurements. The results show that the thermal signal drift of the packaged pressure sensor with a small sensing-channel opening or with a thin silicon membrane for the dam-ring approach had a high packaging induced thermal stress, leading to a high temperature coefficient of span (TCO) response of −0.19% span/°C. The results also show that the thermal signal drift of the packaged pressure sensors with a large sensing-channel opening for sacrifice-replacement approach significantly reduced packaging induced thermal stress, and hence a low TCO response of −0.065% span/°C. However, the packaged pressure sensors of both the sacrifice-replacement and dam-ring type still met the specification −0.2% span/°C of the unpackaged pressure sensor. In addition, the size of proposed packages was 4 × 4 × 1.5 mm^3^ which was about seven times less than the commercialized packages. With the same packaging requirement, the proposed packaging approaches may provide an adequate solution for use in other open-cavity sensors, such as gas sensors, image sensors, and humidity sensors.

## 1. Introduction

In the sensor packaging process, the principal concern is to protect the delicate components of the sensor while retaining a sensing-channel above the silicon membrane. A variety of sensor packaging techniques have been proposed in recent years [1–3]. Pressure sensors have been packaged using a two-step process in which a pre-mold plastic host was embedded within a lead-frame substrate. In an attempt to improve the throughput of the packaging process, while reducing the cost and ensuring compliancy with the SMD process used throughout the electronics and semiconductor fields, researchers have proposed a variety of packaging techniques [4–8] in which the pressure sensor is initially adhered to a lead-frame substrate and then encapsulated using a suitable plastic molding compound. Typical examples of such techniques include the so-called Quad Flat Package (QFP), Dual In-Line Package (DIP), and Small Outline Integrated Circuit (SOIC) packages. However, extra-space has to be reserved for the lead-frame substrates used in these techniques, which inevitably increases the package size.

A key design issue for a pressure sensor is to reserve some free space in a plastic package when a traditional transfer molding approach is adopted. Cotofana *et al*. [9] induced an insert nipple mold to replace the free space of the sensing channel in a plastic package. An inset nipple mold with a gel tip was employed to press against the top surface of the sensor chips in the transfer mold. The insert nipple mold occupied the designed space of the sensing channel, and the free space of the plastic package was finally exposed after the de-molding process. Tseng *et al*. [10] employed soft inset molding technology to partially expose a free space on the die active surface in a molding transferred plastic package. An uncertain contact between the active surface of the chip and the inserted mold produced a defect of EMC bleeding during the transfer molding process. Because the molding compound bleeding contaminated the active surface of the sensor chip, it dramatically affected the yield rate of the packaged sensors. The requirement for new techniques that can be used to fabricate SMD compatible plastic packages and reduce the failure possibility of epoxy molding compound (EMC) bleeding is a major concern in the electronics industry. In our previous developments, a uniformly coating capability of 150 μm with shape keeping ability under extreme molding conditions (165 °C molding temperature/1.86 × 10^6^ Pa molding pressure) for SU-8 photoresist was fabricated [11]. The material residual validation with uniformly coating capability of 150 μm for JSR THB-151N negative-tone photoresist [12] and a plastic packaging of pressure sensors using a SU-8 photoresist [13] were also investigated.

In this work, we focus on the study of tiny plastic packaging with a central opening. Employing a patterned ultra-thick photoresist to pack the MEMS-based pressure sensors, two packaging approaches, sacrifice-replacement and dam-ring, were used. These packaged pressure sensors are fully compliant with the SMD and compatible with batch manufacturing processes. To verify the proposed packaging schemes, a FE model and experimental measurement are used to explore the characteristics of the packaged and unpackaged pressure sensors. The packaging effect and characterization of the packaged pressure sensors for both packaging approaches are conducted and then compared with the performance. The proposed packaging of piezoresistive pressure sensor using a patterned ultra-thick photoresist may be an adequate solution for use in other open-cavity sensors, such as gas sensors, image sensors, and humidity sensors.

## 2. Packaging Design

### 2.1. Packaging Concept

Traditional pressure sensor chips are individually packaged in TO-can or pre-molded housing format, leading to a low packaging throughput and a large packaging body. This study proposes an SMD compatible plastic packaging with an opening sensing-channel, producing a tiny body and high throughput. A photoresist is used to reserve a sensing-channel in the packaging body. According to the same packaging concept, two packaging approaches using two specific photoresist materials are designed. One is called sacrifice-replacement approach and the other is called dam-ring approach. In the sacrifice-replacement approach, a photoresist material is placed just on the top surface of the silicon membrane in a pressure sensor wafer. It is removed from the packaging body after the plastic encapsulation process. In contrast, a photoresist material is deposited enclosing the border of the silicon membrane in a pressure sensor wafer for the dam-ring approach. The deposited photoresist is one part of the whole packaging body, and is not be removed from the packaging body. The function of the photoresists is to protect the sensing channel of the pressure sensor packaging from EMC contamination in a transfer molding process.

### 2.2. Piezoresisive Pressure Sensor

**Figure 1.**
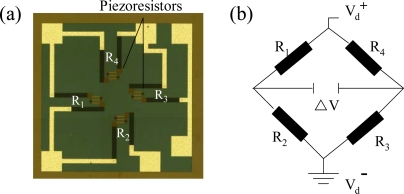
Piezoresistive pressure sensor chip: (a) upper surface showing four piezoresistors; (b) Wheatstone-bridge layout of four piezoresistors.

Conventional micro-machined piezoresistive pressure sensors have a silicon substrate with a back-etched membrane which supports four piezoresistors arranged in such a way that they coincide with the positions of maximum sensitivity for pressure-induced deflections of the diaphragm, as shown in Figure 1(a). A wet etching process is widely used to form the back-etched hollow from the backside of the pressure sensor wafer. To make a closed vacuum chamber in the pressure sensor, the pressure sensor wafer is bonded with a Pyrex glass wafer using an anodic bonding process in a vacuum environment. The back-etched hollow of the pressure sensor and the bonded Pyrex glass, therefore, form a closed vacuum chamber. In practice, the resistance changes of the piezoresistors vary as a function of membrane stress and piezoresistive coefficients of the piezoresistors of the pressure sensor in the longitudinal and transverse directions. The piezoresistive coefficients of piezoresistors vary as a function of both the temperature and doping concentration [14].

Figure 1(b) shows the resistors are electrically connected in a Wheatstone bridge layout. When pressure is applied to the silicon membrane of a pressure sensor, it deflects in the downward direction, causing a change in the resistance values of the four piezoresistors. This in turn induces a change in the output voltage of the Wheatstone bridge. The environment pressure can be derived using the output voltage change of the pressure sensor. The current study considers the package of a p-type silicon pressure sensor deposited in an n-type epilayer with a specific thickness grown on a p-type silicon substrate. The square silicon membrane of the piezoresistive pressure sensor has an area of 576 × 576 μm and a thickness of 20 μm. It is designed for an absolute pressure range of 0–690 × 10^3^ Pa with TCO of 0.2% span/°C.

### 2.3. Packaging Process

#### 2.3.1. Sacrifice-replacement approach

**Figure 2.**
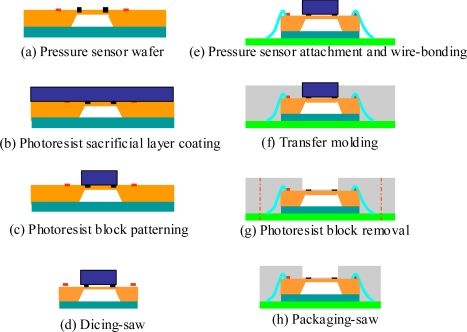
Packaging process of the packaged pressure sensor (sacrifice-replacement approach).

Figure 2 shows the basic packaging steps of the sacrifice-replacement approach. Photoresist materials such as the SU-8 series (Doe Chemical Co., USA), JSR series (JSR Co., Japan), and AZ series (MicroChemical Co., Germany) are widely used in the MEMS field [15–17]. Although the SU-8 series enables the fabrication of structures with characteristic thicknesses ranging from just several tens of micrometers to as much as 2.1mm, a small amount of residual photoresist inevitably remains on the molded structure following the removal process, even when using a highly-effective stripping solution such as hot NMP (1-methy-2-pyrrolidinone) [18]. This residual photoresist is highly undesirable for the pressure sensor package developed in our proposed design, since it randomly affects the original sensing mechanism of the packaged pressure sensor. Tseng and Yu [19] disclosed how the JSR THB-430N was easily removed for the applications of thick-film coating. In contrast to the SU-8 series, the JSR series negative-tone photoresist has far better stripping properties and can generally be removed without leaving any residual trace on the surface. A JSR THB-151N negative tone UV photoresist is a new version of the JSR THB-430N, and it’s also designed for thick-film coating application. Since they hold a similar chemical composition, the THB-151N negative tone UV photoresist is used to be the photoresist block material for the sacrifice-replacement approach.

Initially, a JSR THB-151N negative-tone photoresist (150 μm thick) was spun-coated on the upper surface of a 4 in pressure sensor wafer combined with a Pyrex glass wafer, as shown in Figure 2(b). A traditional photolithographic process was then used to pattern the photoresist layer. The patterned photoresist block covered only the silicon membrane surface of each pressure sensor chip, as shown in Figure 2(c). Figure 2(d) shows a dicing process separated the wafer into multiple individual pressure sensor chips. An adhesive material (Henkel-3880, US) was then dispensed on a die pad of the organic panel substrate (NP-180R, Nan-Ya Plastic Co., Taiwan). The individual pressure sensor chips with a photoresist block were then picked and placed onto the die pads of the organic panel substrate in the form of an *M* × *N* array. Finally, the combined assembly was heated to cross link the adhesive material to bind the pressure sensor chip and the organic panel substrate together. All of the subsequent processing steps can be performed in a batch mode, thus improving the throughput of the packaging process. A wire bonding process was used to electronically couple the signal between the aluminum bonding pads of the pressure sensor chip and the electrode pads of the organic panel substrate, as shown in Figure 2(e). The organic panel substrate was then placed into a transfer mold to encapsulate the pressure sensor chips and the organic panel substrate, as shown in Figure 2(f). The organic panel substrate with EMC encapsulant was placed in a photoresist stripping solution (THB-S1, JSR Co., Japan) to strip the photoresist block from the over-molded package after the de-molding process. The sensing channel of the plastic package was eventually reserved and the silicon membrane surface of the pressure sensor was exposed to interact with atmosphere pressure, as shown in Figure 2(g). After a six hours of post baking at 175 °C to completely cure the molding compound, the individual pressure sensor packages with reserved sensing channels were separated from the over-molded organic panel substrate using a traditional packaging-saw machine (DISCO-DAD-321, Japan), as shown in Figure 2(h). Details of the key packaging procedures are described in Section 2.3.2.

#### 2.3.2. Dam-ring approach

**Figure 3.**
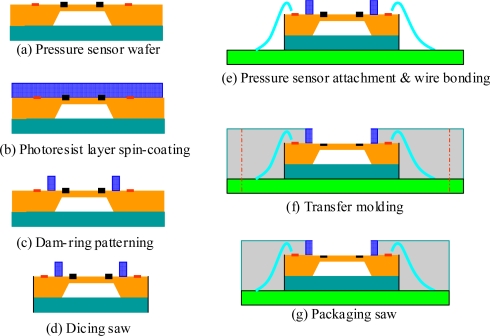
Packaging process of the packaged pressure sensor (dam-ring approach).

Figure 3 shows the basic packaging steps of the dam-ring approach. SU-8 series photoresist has also been used as a low-cost alternative to the LIGA (Lithographic, Galvanoformung, Abformung) process for the fabrication of high-aspect ratio micro-parts and molds [20,21]. Therefore, The SU-8 series photoresist is used to be the photoresist block material for the dam-ring approach.

Initially, an ultra-thick layer (150 μm) of SU-8 negative-tone photoresist is spin-coated on the upper surface of a 4-inch pressure sensor wafer combined with a Pyrex glass wafer. In general, a specific photoresist model is used for a specific coating thickness to achieve a wide operation window. A photoresist with a high viscosity is suitable for high film thickness coating, but the bubble issue during the spin-coating process prevents high yields. Consequently, photoresist models SU8-50 and SU8-100 were used to obtain the coating thicknesses of 50 and 100 μm, respectively. A spin-coater (model SUSS Delta 80BM) and a hotplate (model SUSS Delta 150XBM/T3) were used for the spin-coating process of the dam-ring material. In order to ensure the cleanliness of the wafer surface, the wafer surface was cleaned with acetone, IPA, and DI water before the spin-coating process. A two-stage spin-coating process was employed to produce ultra-thick sacrifice-replacement layer (150 μm). The SU8-100 photoresist was spin-coated after the wafer-cleaning process. The SU8-50 photoresist was then coated on the wafer following a soft baking process of the first coated photoresist layer. The double-layer coating of the photoresist was patterned in a dam-ring shape using the lithographic process. The edge bead removal (EBR) process was used to erase the edge bulge of the photoresist material during each layer coating. Finally, an average coating thickness of 154.9 μm and a coating uniformity of 4.5% were obtained for the two-stage spin-coating process [Figure 3(b)]. Afterwards, a traditional photolithography process was then used to pattern the dam-ring around the silicon membrane surface of the pressure sensors [Figure 3(c)]. A dicing process was employed to separate the wafer into multiple individual pressure sensor chips [Figure 3(d)].

An adhesive material (Henkel-3880, US) was dispensed on die pads of the organic panel substrate. An individual pressure sensor with a dam-ring was then picked and placed onto the die pads of the organic panel substrate by squeezing the adhesive material. Finally, the combined assembly was heated to cross link the adhesive material and to bind the pressure sensor and organic panel substrate together. Subsequent processing steps of the package can be performed in a batch mode, thus improving the throughput of the packaging process. A wire bonding process was carried out to electronically couple the signal between the aluminum bonding pads of the pressure sensors and electrode pads of the organic panel substrate [Figure 3(e)]. Furthermore, the organic panel substrate attached with arrayed pressure sensors was then placed into a transfer-molding mold to encapsulate the pressure sensors and organic panel substrate [Figure 3(f)]. After attaching the individual pressure sensors to the organic panel substrate, the substrate was placed in a transfer mold. This process is an established technique for achieving low-cost encapsulation of electronic products. To minimize thermal-induced warpage of the encapsulated product, the molding compound must be carefully chosen so that its coefficient of thermal expansion (CTE) closely matches that of the organic substrate. In the current study, the pressure sensors were mounted on a FR-5 substrate with a CTE of 12.42 × 10^−6^ K^−1^, and thus an encapsulant with a CTE of 10.0 × 10^−6^ K^−1^ was chosen for molding purposes (ELER-8-700 HS, E’dale Technology Co., LTD, Taiwan). A low molding temperature of 165 °C and a packaging time of 200 seconds were used to eliminate the warping of the whole organic panel substrate. The molding compound and the dam-ring formed a planar surface level following the de-molding process. The sensing channel, which is free space for the interaction between the silicon membrane of the pressure sensor and environment pressure loading, is reserved by the dam-ring. Since the top surface of the dam-ring presses against the inner wall surface of the transfer mold in the mold closed position, the inner space of the dam-ring is not filled by the fluid EMC during the transfer molding process. The sensing channel of the pressure sensor packaging was eventually reserved after the de-molding process. Following the de-molding process, the organic panel substrate with an EMC encapsulant was baked at a temperature of 175 °C for six hours to completely cure the EMC. An individual pressure sensor package with a sensing channel space was separated from the organic panel substrate using a traditional packaging-saw machine after completing the post-curing process of the EMC [Figure 3(g)].

## 3. Finite Element Analyses

### 3.1. Calculation of the Signal Output

In the Wheatstone bridge circuit shown in Figure 1(b), the variation in the output voltage (ΔV) of the pressure sensor was caused by a change in the resistances (*R*_1_, *R*_2_, *R*_3_, and *R*_4_) of the four piezoresistors on the silicon membrane. As shown in Figure 1(a), the four piezoresistors were oriented in the direction of the current flow. Because the resistance change ratio, ΔR/R, for piezoresistor R_1_ is equivalent to that of R_3_ when pressure is applied to the silicon membrane, the resistance change ratio of R_2_ is equivalent to that of R_4_. Using ΔR_1_/R_1_ = ΔR_3_/R_3_ and ΔR_2_/R_2_ = ΔR_4_/R_4_, the variation of the output voltage induced by the silicon membrane deflection is given by:

(1) $$\frac{\Delta V}{V}=1/2\left(\Delta R_{1}/R_{1}-\Delta R_{2}/R_{2}\right)$$

where the R_1_ and R_2_ are referred to as the longitudinal resistance and the transverse resistance, respectively, and *V* is the excitation voltage of the pressure sensor. Based on the concept of the average stress, the real calculated stress location is five micrometers away from the top surface of the silicon membrane. Therefore, the resistance change of each piezoresistor can be computed as:

(2) $$\frac{\Delta R}{R}=\frac{\pi_{l}\;\left(\sum_{i=1}^{n}\sigma_{\mathrm{li}}\;\nu_{i}\right)+\pi_{t}\;\left(\sum_{i=1}^{n}\;\sigma_{\mathrm{ti}}\;\nu_{i}\right)}{\sum_{i=1}^{n}\nu_{i}}$$

where the *i* is an index identifying the piezoresistor element in the FE model, *σ*_l*i*_ and *σ*_t*i*_ are the longitudinal and transverse stresses of the *i*th piezoresistor element, respectively, and *ν_i_* is the volume of the *i*th piezoresistor element. Equations (1) and (2) can be used to calculate the output voltage of the piezoresistive pressure sensor directly from the mechanical stresses induced within each piezoresistor as the membrane deflects.

### 3.2. FE models of the Packaging

**Figure 4.**
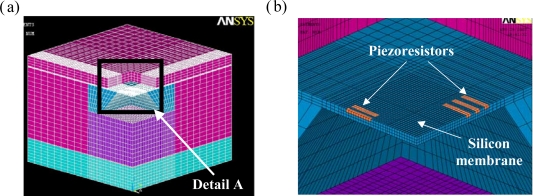
(a) Quarter FE model of the packaged pressure sensor (sacrifice-replacement approach) and (b) a close-up view of detail A.

**Figure 5.**
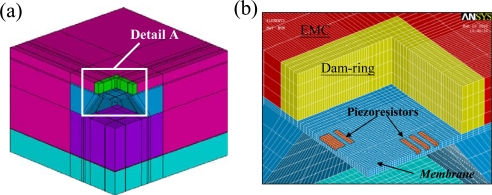
(a) Quarter FE model of the packaged pressure sensor (dam-ring approach) and (b) a close-up view of detail A.

The EMC material employed to package the pressure sensor is a typical epoxy-based polymer, and therefore it shows viscoelastic behavior [22]. Several assumptions are used to develop the FE model. The total stress applied to each piezoresistor is the sum of the average stresses acting on each of the elements within it [23,24]. With the data of dynamic mechanical analysis (DMA) and thermal mechanical analysis (TMA) examinations, the material properties of the EMC are described below. The elastic modulus linearly decreased with temperature from room temperature to 150 °C, and then it dropped dramatically when the EMC was heated up to between 150 °C and 198 °C. Finally, the elastic modulus of the EMC was almost a constant value of 1.5 × 10^9^ Pa when the temperature was over 198 °C. Therefore, the current FE analysis models the EMC as a temperature-dependent linear isotropic material and assumes a stress free temperature at 165 °C (transfer molding temperature). The material properties of the dam-ring are assumed to be isotropic/linear material properties. The package-induced stress is caused by the coefficient of thermal expansion (CTE) mismatch of the packaging materials. In simulating the response of the pressure sensor, the deformation of the piezoresistors is modeled using commercial ANSYS^®^ software (Version 9). Figures 4 and 5 show the mesh structures of the FE models employed for the sacrifice-replacement and dam-ring approaches, respectively.

Due to the inherent symmetry of the pressure sensor packaging, a quarter FE model is sufficient for both dam-ring and sacrifice-replacement approaches. In total, the FE model contains 67,184 eight-node 3D elements, 74,769 nodes, and 222,020 degree of freedom (DOF) for the dam-ring approach. The FE model used for the sacrifice-replacement approach contains 114,912 eight-node 3D elements, 124,527 nodes, and 369,854 DOFs. The boundary conditions of the FE model for both packaging approaches are specified as follows: the *x*-directional displacement is fixed for the *y*–*z* plane, the *y*-directional displacement is fixed for the *x*–*z* plane, and the center node of the lower surface of the organic substrate is fixed in the *x-*, *y*-, and *z-* directions.

## 4. Results and Discussion

### 4.1. Packaged Pressure Sensors

#### 4.1.1. Sacrifice-replacement approach

**Figure 6.**
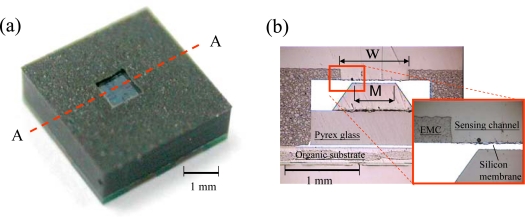
(a) 3D image of the packaged pressure sensor (sacrifice-replacement approach) and (b) its cross-section image at section A-A.

Figure 6 shows the exterior appearance of the packaged pressure sensor (sacrifice-replacement approach) and its cross-section image at section A-A. The top surface of the sensing membrane was entirely free of EMC contamination, so the effectiveness of the photoresist block in shielding the silicon membrane of the pressure sensor was confirmed. The top surface of the silicon membrane was fully exposed to freely sense the environment pressure through the sensing-channel of the packaged pressure sensor. To ensure a full contact between the mold surface and all of the sacrificial photoresist blocks, a very small interference value was deliberately designed between the depth of the mold cavity and the total package height. Consequently, the top surface of the photoresist block was squeezed as the transfer mold was closed. The concave appearance of the photoresist block was due to the mold squeeze and can be improved with a proper interference design. The photoresist block also effectively protects the sensing membrane of the pressure sensor from EMC bleeding, improving the yield of the packaging process. As shown in Figure 6(b), the sidewalls of the sensing channel tapered slightly in the outward direction. The opening size of the sensing channel on the topside is slightly larger than that on the bottom-side. The slight geometry deformation in the cross-section of the sensing-channel has no negative influence on the pressure sensing of the packaged pressure sensor. The sacrifice-replacement approach was verified as useful for a pressure sensor application. Due to the process requirement of cold-mount specimens, a clear epoxy (CMA1-K02, Pentad Scientific Co., Taiwan) was used to fill the cold-mound mold during the specimen preparation process. The original space of the open channel is a real cavity instead of the cold-mounted clear epoxy, as shown in the cross-section image of the packaged pressure sensor [Figure 6(b)].

#### 4.1.2. Dam-ring approach

**Figure 7.**
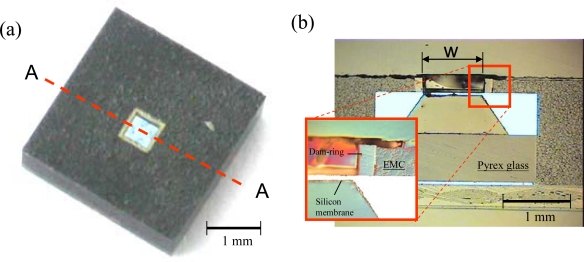
(a) 3D image of the packaged pressure sensor (dam-ring approach) and (b) its cross-section image at section A-A.

Figure 7 shows three-dimensional (3D) and cross-section images of the central region of the pressure sensor package assembled with the dam-ring approach. Free space for the sensing-channel was completely reserved in the central area of the plastic pressure sensor package. The top surface of the silicon membrane was free of EMC contamination, as shown in Figure 7(b). Therefore, the dam-ring parts successfully shields the inner space of the dam-ring from the overflowing or leakage of fluid EMC at a high molding temperature of 165 °C. The injected molding pressure pushed the fluid EMC which squeezed the outer sidewall of the dam-ring during transfer molding process so that the peripheral borders of the dam-ring had a slight inward deformation. However, the dam-ring was still tightly bound to the top surface of the pressure sensor. The fluid EMC did not bleed to cover the top surface of the silicon membrane through the interface between the dam-ring and the top surface of the pressure sensor. Therefore, the dam-ring performed its function. The slight deformation of the dam-ring walls can be improved by employing a thicker dam-ring sidewall to enhance its bending strength. The packaging dimensions of the packaged pressure sensors, sacrifice-replacement and dam-ring approaches, are 4 × 4 × 1.5 mm^3^ and Table 1 shows the basic dimensions of the both packaged pressure sensors. Table 2 shows the dimension comparisons between our proposed two packaged pressure sensors and the commercialized pressure sensor packages. Both packaging volumes of the proposed two packaged pressure sensors are about seven times less than the commercialized pressure sensor packages.

### 4.2. Validation of the FE Models

**Figure 8.**
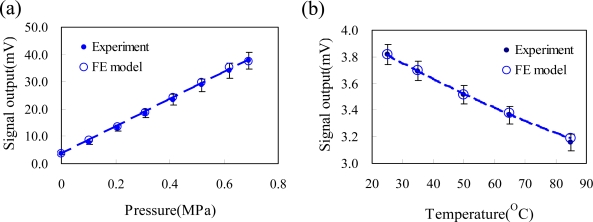
Output voltage of the unpackaged pressure sensor (a) as a function of the pressure at 25 °C and (b) as a function of the temperature at 0 Pa for the FE model and experiment results.

To validate the FE model, the measured signal output of an unpackaged pressure sensor was used to compare with calculated signal output of FE model. Figure 8 shows the FE model and experimental results of the unpackaged pressure sensor under a pressure loading range of 0–6.9 × 10^3^ Pa and a temperature loading range of 25–85 °C. The FE model closely matches the experimental data, as shown in Figure 8. The average error between the FE model and the experimental result was less than 4.2%. The measured results of the unpackaged pressure sensor were collected from five samples. Due to the doping impurity variation of the piezoresistors, the four piezoresistors of the piezoresistive pressure sensor had different resistances. The reference pressure in the cavity of the pressure sensor was vacuum for an absolute pressure sensor, so an initial 1 atmosphere (about 101.3 × 10^3^ Pa) pressure was applied on the silicon membrane of the pressure sensor at pressure loading of 0Pa. The zero offset voltage (Δ*V*) of each pressure sensor was a non-zero value according to Equation 1, and it varied from sensor to sensor. However, the resistances of the four piezoresistors of the pressure sensor are assumed to be equal in the FE modeling process. The calculated zero offset value of the FE model was calculated using a unique ideal sensor chip. This explains why the zero offset voltage of the experimental measurements is different from that of the FE model results. The FE model was verified to reliably predict the signal output of the piezoresistive pressure sensor under pressure and temperature loading.

**Figure 9.**
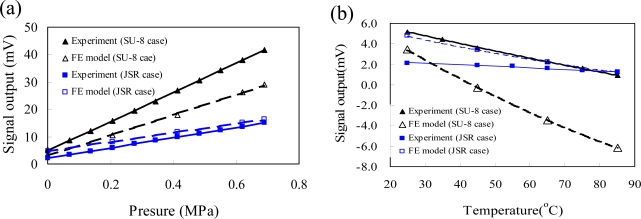
Output voltage of the packaged pressure sensor (a) as a function of the pressure at 25 °C and (b) as a function of the temperature at 0 Pa for the FE model and experiment

Figure 9 shows the calculated and measured output voltages of the packaged pressure sensors in a pressure range of 0–6.9 × 10^3^ Pa and a temperature range of 25–85 °C. The data labels of symbol “SU-8 case” and “JSR case” are used to represent the result of the packaged pressure sensor using dam-ring and sacrifice-replacement approaches, respectively. In Figure 9(a), the calculated response curve of the FE model well agrees with measured response curve of the packaged pressure sensor for both packaging approaches. As shown in Figure 9(b), there is a discrepancy between the FE model and the experimental results for both packaging approaches. The FE model assumes that the package-introduced stress is mostly from the CTE mismatch of packaging components. The discrepancy could have resulted from the non-symmetrical placement of the pressure sensor in the packaging process, since the different stress distributions on the silicon membrane of the pressure sensor change the final output voltage of the pressure sensor. The final material properties of the EMC are strongly dominated by the final curing situation. In addition, a simplified linear model could also contribute to the discrepancy [25,26]. However, the signal outputs of the packaged pressure sensor obtained from the FE model and the experiment have similar trend. In addition, the thickness variation of the silicon membrane is ±10% for a commercial specification of the piezoresistive pressure sensors. This induces a shift on zero-offset output for pressure sensors. Therefore, the zero offset voltage (Δ*V*) of each pressure sensor is a non-zero value and varies from sensor to sensor. To set the signal output of a pressure sensor always on a positive voltage output at all pressure and temperature range, a series resistor is added on the sensing circuit to shift the zero-offset output of the pressure sensor to a specified positive zero-offset value. In contrast, the output voltage of the pressure sensor is caught directly from the output of the Wheatstone bridge (without adding the series resistor). This explains why the signal output of the pressure sensor may cover a range of positive and negative voltages.

### 4.3. Characterization

#### 4.3.1. Packaging effect

**Figure 10.**
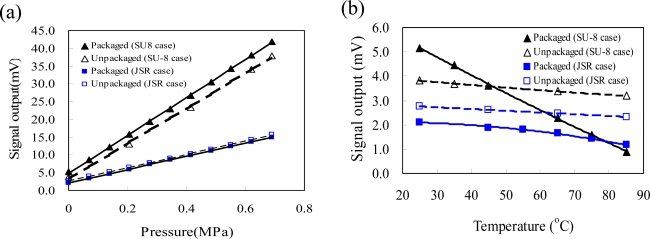
Measured output voltage of unpackaged and packaged pressure sensors (a) as a function of the pressure at 25°C and (b) as a function of the temperature at 0 Pa.

To investigate the packaging effect on the sensing characteristics of the pressure sensor, characterization comparisons between unpackaged pressure sensor and packaged pressure sensors were conducted for both packaging approaches. Due to the different manufacturing lots of the pressure sensor wafers, the silicon membrane thickness of the pressure sensors are 20 and 30 μm for the dam-ring and sacrifice-replacement approaches, respectively. Figure 10 shows the signal output comparison between the unpackaged and packaged pressure sensors under various temperature and pressure loadings. A signal output difference between the packaged and the unpackaged pressure sensors is observed, as shown in Figure 10(a). Due to the shrinkage characteristics of the molding compound material during the post-molding curing process, a silicon membrane surface of the pressure sensor has a compression stress distribution, inducing a signal output shift. Besides, the original resistance of the four piezoresistors varies from chip to chip. That is one of the possible causes of the signal output shift of the packaged pressure sensor. However, the packaged pressure sensor has a similar response trend with that of the unpackaged pressure sensor at room temperature.

As shown in Figure 10(b), the output voltage decreases when the temperature increases. The packaging exerts a thermo-mechanical stress on the silicon membrane of the pressure sensor and induces a higher thermal voltage shift compared with the unpackaged pressure sensor. TCO is a sensing feature to estimate the sensing stability for a pressure sensor operated in a temperature condition range under a specific pressure loading. The specific pressure loading indicated is 0Pa loading and TCO can be calculated from the equation 3. The measured TCO is −0.03% span/°C for an unpackaged pressure sensor and is −0.19% span/°C for a packaged pressure sensor with dam-ring approach. The packaging induced thermal stress dominated the thermal signal drift of the packaged pressure sensor for the dam-ring approach:

(3) $$\text{TCO}=\left(\Delta V_{t}/V_{r}\right)/\Delta T$$

where the ΔV_t_ is the signal output variation range of pressure sensor under temperature variation range (ΔT) at 0Pa and V_r_ is signal output of the pressure sensor at room temperature and 0 Pa.

**Figure 11.**
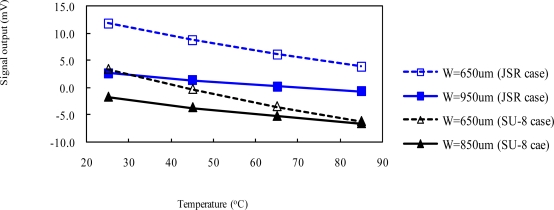
Calculated output voltage as a function of the temperature at 0 Pa for the packaged pressure sensors with various sensing channel openings.

Since the thermo-mechanical stress of the packaging is mostly due to the CTE mismatch of the packaging materials [27], enlarging the clearance between EMC and the border of the silicon membrane can reduce the thermal stress effect. Packaged pressure sensor with a large value of the sensing-channel opening, symbol “W” indicated in Figures 6(b) and 7(b), means a large clearance between EMC and the border of the silicon membrane. Therefore, packaged pressure sensors with various sensing-channel openings were evaluated using the verified FE model to explore the issue of thermal voltage drift for both packaging approaches. Figure 11 shows the output voltage of packaged pressure sensors with various sensing channel openings as a function of the temperature at 0 Pa. The calculated results show that the packaged pressure sensors with a large sensing-channel opening strongly reduce the thermal signal drift resulting from the package-induced thermal stress. It is helpful on the packaging design of the proposed packaging approaches. Because the sensing-channel openings of the fabricated pressure sensor packaging are 650 and 950 μm for the dam-ring and sacrifice-replacement approaches, respectively, it also explains why the thermal signal drift of the packaged pressure sensor for the dam-ring approach is stronger than that for the sacrifice-replacement approach, as shown in Figure 10(b).

**Figure 12.**
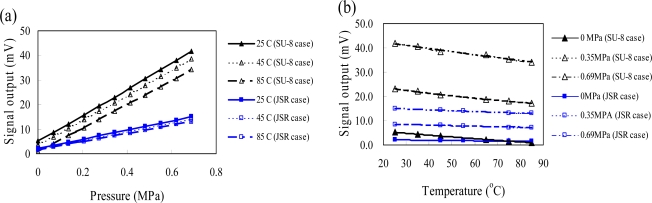
Measured output voltage of the packaged pressure sensor (a) as a function of the pressure and (b) as a function of the temperature.

Figure 12 shows signal outputs of the packaged pressure sensors for both packaging approached at a temperature range of 25–85 °C and a pressure range of 0–690 kPa. In general, a pressure sensor with a thin silicon membrane has a more sensitive response than that with a thick silicon membrane. The sensing-channel opening value for a dam-ring approach is smaller than that of the sacrifice-replacement approach. The thermal signal drift is more notable for the dam-ring approach due to the small sensing-channel opening [Figure 12(a)]. TCO is independent with the applied pressure range for a piezoresistive pressure sensor, since the piezoresitive coefficients of the piezoresistors are function of temperature. As shown in Figure 12(b), TCOs of the packaged pressure sensors are −0.19 and −0.065% span/°C at 0 Pa for dam-ring and sacrifice-replacement approaches, respectively. It is clear that the packaged pressure sensor with a small sensing-channel opening or with a thin silicon membrane has a high packaging induced thermal stress, leading to a high TCO characteristic. The thermal signal drift of the packaged pressure sensors with a large sensing-channel opening for sacrifice-replacement approach significantly reduced packaging induced thermal stress. However, both packaged pressure sensors meet the specifications of the unpackaged pressure sensor (−0.2% span/°C).

**Figure 13.**
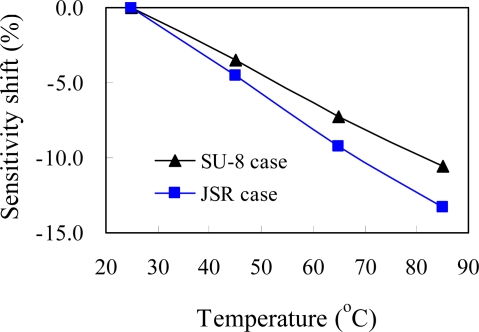
Sensitivity shift of the packaged pressure sensors as a function of the temperature at 0 Pa.

Sensitivity shift is the temperature variation of the sensitivity and is defined as:

(4) $$\text{Sensitivity shift}\;=\left(S_{t0}-S_{t}\right)/S_{t0}$$

where S_t0_ is the sensitivity at the reference temperature and S_t_ is the sensitivity at ambient temperature t. In this work, the reference temperature is the room temperature. The sensitivity shift feature of the two packaged pressure sensors fabricated by sacrifice-replacement and dam-ring approaches are carried out and the sensing channel openings for both packaged pressure sensors are 950 and 650 μm, respectively. Figure 13 shows the sensitivity drift of the packaged pressure sensor as a function of temperature for both packaging approaches. The packaged pressure sensors have a linear sensitivity shift over temperature range of 25–85 °C for the proposed packaging approaches, making an easily and low costly on the post control circuits design.

#### 4.3.2. Comparison of packaging approaches

Using an ultra-thick photoresist, two packaging approaches, sacrifice-replacement and dam-ring, are developed referring to an identical packaging concept. The patterned photoresist for dam-ring approach is one part of the final packaged pressure sensor. In contrast, the patterned photoresist for sacrifice-replacement approach is temperately deposited in the packaging body, and it has to been removed from the packaged pressure sensor. The dam-ring approach eliminates an extra removal process of the completely cured photoresist, improving the packaging throughput compared with the sacrifice-replacement approach. In general, to remove a photoresist with completely cured is more difficult than that with just soft-baked. In comparison with sacrifice-replacement approach, the dam-ring approach is suitable for application of other open-cavity sensors, such as gas sensors. Because the photoresist residual on the sensing material layer of the gas sensors will reduce the effective sensing surface area and degrade the sensing performance at the same time. In this study, the sensing feature, the packaged pressure sensor using the dam-ring approach has a similar packaging feature compared with that using the sacrifice-replacement approach, if the same sensing channel opening is designed. However, the photoresist used for sacrifice-replacement approach just covers the sensing active area of the sensor chips only, such as the silicon membrane area for a pressure sensor. The sacrifice-replacement approach is suit for the sensor chip having small total chip size and large sensing active area.

## 5. Conclusions

This study demonstrated a novel pressure sensor packaging using patterned ultra-thick photoresists. The photoresist materials used for both sacrifice-replacement and dam-ring approaches can prevent the sensing-channel of the pressure sensor packaging from EMC contamination under molding transfer conditions of 165 °C and 1.86 MPa. The thermal signal drift of the packaged pressure sensors with a large sensing-channel opening for sacrifice-replacement approach significantly reduced packaging induced thermal stress, and hence a low TCO response of −0.065% span/°C. Both packaged pressure sensors of sacrifice-replacement and dam-ring approaches still met the specification −0.2% span/°C of the unpackaged pressure sensor. In addition, the size of proposed packages was 4 × 4 × 1.5 mm^3^ which was about seven times less than the commercialized packages. With the same packaging requirement, the proposed packaging approaches can be an adequate solution for use in other open-cavity sensors, such as gas sensors, image sensors, and humidity sensors.

## Figures and Tables

**Table 1.** Packaging dimensions of the packaged pressure sensors.

**Items Unit**		**Packaging dimensions**	
		**Sacrifice-replacement approach**	**Dam-ring approach**
Packaging size	mm^3^	4.0 × 4.0 × 1.5	4.0 × 4.0 × 1.5
Sensing channel opening (w)	μm	950	650
Sensing channel depth	μm	150	150
Silicon membrane size (h)	μm^2^	576 × 576	576 × 576
Silicon membrane thickness	μm	20	30

**Table 2.** Packaging volume comparisons between proposed packaged pressure sensors and the commercialized pressure sensor packages.

	**Freescale (MPXHZ6130)**	**Metrodyne (MPS-3100)**	**BOSCH (SMD085)**	**NSYSU (this study)**
	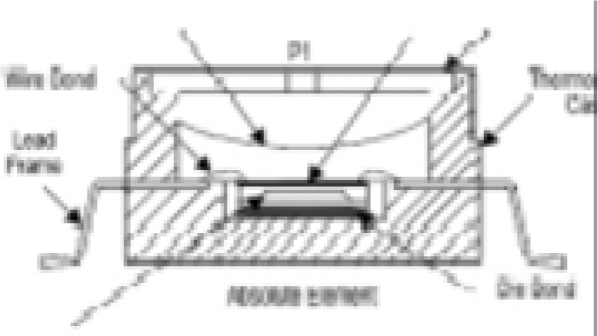	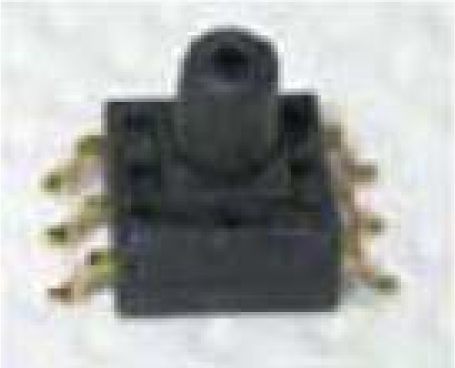	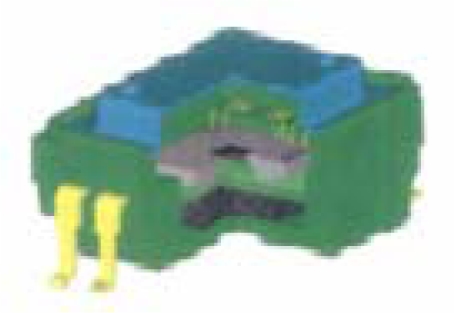	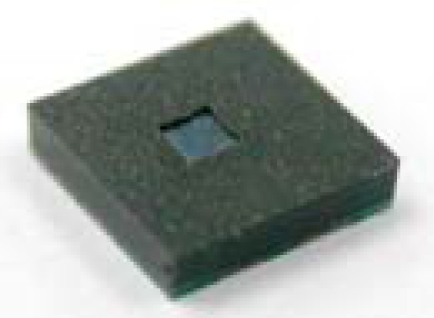
**Packaging size (mm)**	7.4 × 7.4 × 3.9	7.0 × 7.0 × 4.0	8.4 × 6.9 × 4.6	4.0 × 4.0 × 1.5
**Packaging volume**	7.8	7.0	8.8	1.0*

## References

[1] Janczek T. Material investigation for pressure sensor package p-dsof-8-1.

[2] Abbaspour-Santi E., Afrang S., Teymoori M.M. A novel method for packaging of micromechined piezoresistive pressure sensor.

[3] Bitko G., Monk D.J., Maudie T., Stanerson D., Wertz J., Matkin J., Petrovic S. Analytical techniques for examining reliability and failure mechanism of barrier coating encapsulated silicon pressure sensor exposed to harsh media.

[4] Nyather J.B., Larsen A., Liverod B., Ohlckers P. (1998). Measurement of package-induced stress and thermal zero shift in transfer molded silicon piezoresistive pressure sensors. J. Micromech. Microeng.

[5] Dancaster J., Kim W., Do D. Two-chip pressure sensor and single conditioning.

[6] Krondorfer R., Kim Y.K., Kim J., Gustafson J.K., Lommasson T.C. (2004). Finite element simulation of packaging stress in transfer molded MEMS pressure sensors. Microelectron. Reliab.

[7] Campabadal F., Cmeras L., Arrieta M.J. Packaging of silicon pressure sensors for home application.

[8] Krondorfer R., Kim Y.K. (2007). Packaging effect on MEMS pressure sensor performance. IEEE Trans. Compon. Packag. Technol.

[9] Cotofana C., Bossche A., Kaldenberg P., Mollinger J. (1998). Low-cost plastic sensor packaging using the open-window packaging concept. Sens. Actuat. A: Phys.

[10] Tseng H.K.E, Zong Z.B., Lee S.G., Ho S.C., Srikanth N. Development of transfer molding technology for package with die active side partially exposed.

[11] Hsu C.Y., Chen L.T., Chang J.S., Chu C.H. A novel packaging process for open-channel sensors.

[12] Chen L.T., Hsu C.Y., Chang J.S., Shieh W.L., Xie Y.Z., Chu C.H. A tiny plastic package of piezoresistive pressure sensors constructed by sacrifice-replacement approach.

[13] Chen L.T., Cheng W.H. (2009). A novel plastic package for pressure sensors fabricated using the lithographic dam-ring approach. Sens. Actuat. A: Phys.

[14] Smith C.S. (1954). Piezoresistance effect in germanium and silicon. Phys. Rev.

[15] Roth S., Dellmann L., Racine G.A., Rooij N.F. (1999). High aspect ratio UV photography for electroplated structure. J. Micromech. Microeng.

[16] Brunet M., O’Donnell T., O’Brien J., McCloskey P., Mathuna S.C.Ó. (2002). Thick photoresist development for the fabrication of high aspect ratio magnetic coils. J. Micromech. Microeng.

[17] Kukharenka E., Kraft M. (2003). Reliability of electroplating mold with thick positive spr 220-7 photoresist. J. Mater. Sci. Mater. Electron.

[18] Despont M., Lorenz H., Fahrni N., Brugger J., Renaud P., Vettiger P. (1997). High-aspect-ratio ultrathick negative-tone near-UV photoresist and its applications for MEMS. J. Micromech. Microeng.

[19] Tseng F.G., Yu C.S. (2002). High aspect ratio ultra-thick micro-stencil by JSR THB-430N negative tone UV photoresist. Sens. Actuat. A: Phys.

[20] Lee K.Y., LaBianca N., Rishton S.A., Zolgharnain S., Gelorme J.D., Shaw J., Chang T.H.-P. (1995). Micromachining applications of a high resolution ultratick photoresis. J. Vac. Sci. Technol. B.

[21] Lorenz H., Despont M., Vettiger P., Renaud P. (1998). Fabrication of photoplastic high-aspect ratio micro-parts and micro-molds using SU-8 UV resist. Microsyst. Technol.

[22] Tsai M.Y., Wang C.T., Hsu C.H. (2006). The effect of epoxy molding compound on thermal/residual deformations and stress on IC packaging during manufacturing process. IEEE Trans. Compon. Packag. Technol.

[23] Lee C.C., Peng C.T., Chiang K.N. (2006). Packaging effect investigation of CMOS compatible pressure sensors using flip chip and flex circuit board technologies. Sens. Actuat. A: Phys.

[24] Peng C.T., Lin J.C., Lin C.T., Chiang K.N. (2005). Performance and package effect of a novel piezoresistive pressure sensor fabricated by front-side etching technology. Sens. Actuat. A: Phys.

[25] Krondorfer R., Kim Y.K. (2007). Packaging effect on MEMS pressure sensor performance. IEEE Trans. Compon. Packag. Technol.

[26] Xu J., Zhao Y., Jiang Z. Analysis of the packaging stress in monolithic multi-sensor.

[27] Nysether J.B., Larsen A., Liverod B., Ohlckers P. (1998). Measurement of package-induced stress and thermal zero shift in transfer molded silicon piezoresistive pressure sensors. J. Micromech. Microeng.

